# The effect of surface morphology on endothelial and smooth muscle cells growth on blow-spun fibrous scaffolds

**DOI:** 10.1186/s13036-021-00278-1

**Published:** 2021-12-19

**Authors:** Iwona Łopianiak, Michał Wojasiński, Aleksandra Kuźmińska, Paulina Trzaskowska, Beata A. Butruk-Raszeja

**Affiliations:** 1 Laboratory of Biomedical Engineering, Faculty of Chemical and Process Engineering, Warsaw University of Technology, Waryńskiego 1, 00-645 Warsaw, Poland; 2 Centre for Advanced Materials and Technologies CEZAMAT, Warsaw University of Technology, Poleczki 19, 02-822 Warsaw, Poland

**Keywords:** One-layer vascular graft, Bi-layer vascular graft, Solution blow spinning, Polyurethane, Endothelial cells, Smooth muscle cells

## Abstract

This study aimed to analyze the growth of two types of blood vessel building cells: endothelial cells (ECs) and smooth muscle cells (SMCs) on surfaces with different morphology. Two types of materials, differing in morphology, were produced by the solution blow spinning technique. One-layer materials consisted of one fibrous layer with two fibrous surfaces. Bi-layer materials consisted of one fibrous-solid layer and one fibrous layer, resulting in two different surfaces. Additionally, materials with different average fiber diameters (about 200, 500, and 900 nm) were produced for each group. It has been shown that it is possible to obtain structures with a given morphology by changing the selected process parameters (working distance and polymer solution concentration). Both morphology (solid versus fibrous) and average fiber diameter (submicron fibers versus microfibers) of scaffolds influenced the growth of ECs. However, this effect was only visible after an extended period of culture (6 days). In the case of SMCs, it was proved that the best growth of SMCs is obtained for micron fibers (with an average diameter close to 900 nm) compared to the submicron fibers (with an average diameter below 900 nm).

## Introduction

Cardiovascular diseases (CVDs), classified as civilization diseases, are currently the major cause of death globally [1]. Among them, coronary heart disease and peripheral arterial disease are the most common and dangerous for human life [2]. In advanced stages, these diseases lead to heart attacks or strokes caused by the complete clogging of the blood vessels. Coronary artery bypass grafting is one of the treatment methods of advanced ischemic heart disease. Patients’ autologous blood vessels (e.g., saphenous veins) are currently the most frequently used as bypasses [3, 4]. However, in many cases, the poor condition of the patient’s vein makes it impossible to use for transplant. Moreover, there are problems with the long-term patency of the transplanted blood vessel, which necessitates the need for further surgical interventions [5, 6]. The low availability of autologous blood vessels and problems after implantation generate the need to look for other solutions to save patients’ health and life [7]. An alternative solution is synthetic polymer prostheses that imitate the structure, functions, and properties (mechanical and morphological) of native vessels [8, 9]. Meaning that prostheses’ porous structure and appropriate mechanical properties should provide proper conditions for cells to infiltrate the prosthesis, proliferate and grow [10, 11].

Two types of cells: endothelial cells (ECs) and smooth muscle cells (SMCs), build native blood vessels. Mimicking the blood vessel layered structure is an essential aspect during vascular prostheses designing. The inner layer should promote the formation of the endothelium, enable oxygen and nutrients transport, and prevent the migration of cells through this layer. In contrast, the outer layer should allow the migration of SMCs inside the prosthesis structure [12].

Previous studies show that among the numerous factors determining the proper development and activity of cells, one of the most important is the surface morphology. In the case of fibrous materials, the fiber diameter and the pore size seem to be crucial. According to the results presented by other authors, fibers with diameters <1 μm are optimal for endothelial cells' growth. Ju et al. showed that endothelial cells growing on nanofibers (approx. fiber diameter = 270 nm) presented numerous actin fibers and formed stronger focal adhesion contacts than those growing on microfibers [12]. Similar results were presented by others [13]. The diameter of the fibers also influences the development of SMCs. It has been shown that increasing fiber diameter reduced SMC proliferation and increased SMC infiltration [14]. In turn, studies have shown that reducing the diameter of the fibers accelerates the proliferation and maturation of SMCs [15]. Finally, pore size is crucial parameter during neotissue formation[16]. The answer to these diverse needs is the creation of layered prosthesis in which the properties of the inner and outer layers are different, in order to better support the development of a specific type of cells.

Work by Goins et al. review techniques used in the production of layered vascular prostheses [17]. Techniques for fabrication of layered prosthesis are mainly based on electrospinning (ES) [18–21]. In addition, various processes combining ES and other techniques have been proposed: melt electrowriting [22], knitting [23], phase separation [24]. Compared to ES, solution blow spinning (SBS) technique is distinguished by several advantages, i.e. a simpler system, not requiring the use of electric voltage, and higher production efficiency, which is especially important in the case of multi-layer structures production with a thickness of up to several hundred micrometers. So far, it has been proposed to modify the internal prosthesis by applying the blow spun fibers [25]. Also, layered prosthesis produced by SBS combined with dip spinning [26] were produced.

In this paper, one- and bi-layer fibrous materials with different fiber diameters were fabricated using only one technique - SBS. In contrast to the above-mentioned works, based on ES or mixed techniques, we propose a simple SBS-based process that allows to control the critical parameters of the manufactured prosthesis, i.e. the average diameter of the fibers, the number and thickness of layers in the prosthesis’ wall. Obtaining the appropriate product is possible thanks to the change of basic process parameters, i.e. working distance, concentration of the polymer solution. The presented technology is the subject of a patent. Medical-grade polyurethane was used as a polymer of choice, due to its relatively high hemocompatibility.

Three groups of materials differing in average fiber diameter (about 200, 500 and 900 nm) and two groups of materials with different structures (one-layer and bi-layer) were produced. One-layer (1L) scaffolds consisted of one fibrous layer. Bi-layer (2L) scaffolds consisted of one fibrous-solid layer and one fibrous layer. After characterizing the physical and mechanical properties of the obtained structures, the influence of surface morphology on the growth of ECs and SMCs was examined. ECs were seeded on the surface marked as inner (IS, collector side). The opposite surface, labeled as the outer (OS), served as the surface for SMCs growth. The analysis of cell growth allowed for selecting the most favorable surfaces for the growth of both types of cells, which will allow for the appropriate design of the structure of the blow-spun vascular prosthesis.

The main goal of this study was to analyze the various morphological types of surfaces obtained with the SBS technique. In particular, we focused on the influence of morphology on the growth of ECs and SMCs. The presumed result was the selection of materials that present high coverage of ECs and high infiltration of SMCs. The selected surfaces will enable further, more detailed work, i.e. cell cultures in flow, analysis of specific cell activity markers.

## Materials and methods

### Scaffold fabrication

Polyurethane (ChronoFlex C75A, AdvanSource Biomaterials) nano/microfibrous materials in the form of cylindrical scaffolds were produced in the SBS process, described in detail elsewhere [27]. Here, 1,1,1,3,3,3-hexafluoro-2-propanol (>99.0%, TCI Chemicals) was used as a solvent. Polymer solutions were prepared overnight in concentrations of 2, 4, and 5% w/w. To produce nano/microfibrous materials, each polyurethane solution was supplied through the inner nozzle of the concentric nozzles system in the SBS apparatus with a constant flow rate of 30 ml/h. Simultaneously, the airstream was supplied through the outer nozzle of the SBS nozzles system with a pressure of 0.1 MPa. Fibers were produced by shear-drag elongation of the polymer solution by the stream of air on the distance between the nozzles system and the surface of the collector (working distance). Nano/microfibrous materials were prepared as one-layer materials and bi-layer materials. One-layer scaffolds were produced using a 30 cm working distance. Bi-layer scaffolds were produced by changing working distance during the process. The first layer was produced using a 10 cm working distance and by blowing 20% of the total polymer solution volume. The second layer was produced using a 30 cm working distance and by blowing 80% of the total polymer solution volume. The volume of polymer solution for the scaffold production was adjusted to fabricate cylindrical scaffolds with wall thickness in the range from 300 to 500 μm. Detailed process parameters are presented in Table 1. The rotating cylinder (cylinder diameter: 3 mm, length: 120 mm, rotational speed: 3 000 rpm) was used as a collector. Cylindrical samples were pulled off the collector and kept in ventilated containers overnight to ensure complete solvent evaporation. For microscopic analyzes and cell culture, cylindrical samples were cut open and flattened. The inner surface of the cylinder (from the collector side) was marked as IS, while the opposite outer surface was marked as OS.

**Table 1:** Parameters of the SBS process applied during material fabrication. The numbers in brackets correspond to the volumes of polymer solution used to produce the first and the second layer in bi-layer scaffolds

Sample	Polymer solution concentration [% w/w]	Number of layers [-]	Polymer solution volume (1st layer/2nd layer) [ml]	Working distance (1st layer/2nd layer) [cm]
C75A_1L_200	2	1	20	30
C75A_1L_500	4	1	5	
C75A_1L_900	5	1	5	
C75A_2L_200	2	2	20 (4/16)	10/30
C75A_2L_500	4	2	5 (1/4)	
C75A_2L_900	5	2	5 (1/4)	

### Scaffold characterization

#### Fiber diameter, pore size

Rectangular samples were subjected to scanning electron microscopy (SEM, Phenom G1, PhenomWorld). Samples were coated with a 15 nm layer of gold/palladium alloy (80/20 at%) using a sputter coater (K550 Emitech, Quorum Technologies). Ten randomly selected spots were photographed with 5000x magnification, and the images were used for fiber diameter and pore size measurements. All measurements were performed using Fiji software [28]. Results are presented as fiber size distributions (*n *= 100), mean fiber diameter, standard deviation, minimum and maximum fiber diameter. Pore sizes were measured using the same images, and mean pore size (*n *= 100) ± standard deviation is reported. Microscopic analysis was performed for both surfaces of cylindrical scaffolds (OS and IS).

#### Surface wettability

Materials in the form of cylinders were cut open to obtain flat mats. Both the inner (IS) and outer (OS) surfaces were subjected to wettability analysis. Samples were glued to a glass slide, and a drop of distilled water (5 µl) was placed on a clean and dry surface. The contact angle was measured automatically using Kruss DSA 100 software; the measurement was performed in at least 10 randomly selected spots on analyzed material. Each material variant was tested in triplicate (*n *= 30).

#### Porosity

The porosity of all types of polyurethane cylindrical scaffolds was measured using the gravimetric method, described in detail elsewhere [29]. In general, the thickness of each sample (*n *= 25) was measured based on the SEM images of scaffolds cross-section (*n *= 5), and then the volume of the sample (V_s_) was calculated (inner diameter of the scaffold was 3 mm). Each sample (*n *= 5) was weighted (m_s_), and the apparent density (ρ_app_) for all samples was calculated using the following equation: ρ_app_ = m_s_ ∙ V_s_^−1^ [g/cm^3^]. Then, the scaffold porosity (ε, *n *= 5) was calculated using the following equation: ε = 1 - ρ_app_ ∙ ρ_p_^−1^, where ρ_p_ – polyurethane density (1.2 g/cm^3^) [30].

#### Mechanical properties

Cylindrical samples (3 mm inner diameter, 70 mm length) of fibrous scaffolding materials underwent a uniaxial stretching test according to protocols established based on ASTM standards (Designation: D 882-02 and D 638-02a). The experiment was conducted using an Instron 3345 model with pneumatic jaws within 50 mm of each other. The 10 mm long tips of samples were placed in the pneumatic jaws of the testing machine, so that the central part of the sample (50 mm length) was stretched with crosshead speed 5 mm ∙ min^−1^ at room temperature and humidity. Load-strain curves were recorded, as were the maximum load and strain at rupture. It is emphasized that according to porosity measurements and SEM images, only a fraction of each sample thickness is occupied with fibers (1-ε), which implies that the applied load is supported by only such a fraction of the sample’s thickness. This effect was accounted for in the data processing for maximum stress calculation. For each type of scaffold, results of Young's modulus, elongation at break, and tensile strength are presented as mean values ± standard deviation (*n *= 5).

### Cell culture

All materials before culture were placed in a 1% v/v antibiotic/antimycotic solution (100 U/ml penicillin G, 100 µg/ml streptomycin sulfate, and 0.25 µg/ml amphotericin B) diluted in sterile phosphate-buffered saline (PBS) for 24 h at 4 °C for sterilization. Then, scaffolds were washed three times with sterile deionized water on a roller for 5 min each time.

ECs (ATCC) were cultured at 37 °C in a 5% CO_2_ humidified atmosphere using MCDB-131 medium with phenol red and supplemented with 10% fetal bovine serum (ATCC), 1% penicillin-streptomycin (Gibco), 10 mM L-glutamine (Gibco), 1 µg/ml hydrocortisone (Sigma-Aldrich) and 10 ng/ml endothelial growth factor (Life Technologies).

SMCs (Lonza) were cultured in an incubator (37 °C, 5% CO_2_ humidified atmosphere). Cells were grown in Smooth Muscle Cell Growth Medium 2 (PromoCell), supplemented with fetal calf serum (0.05 ml/ml), epidermal growth factor (0.5 ng/ml), basic fibroblast growth factor (2 ng/ml), insulin (5 µg/ml).

**Fig. 1:**
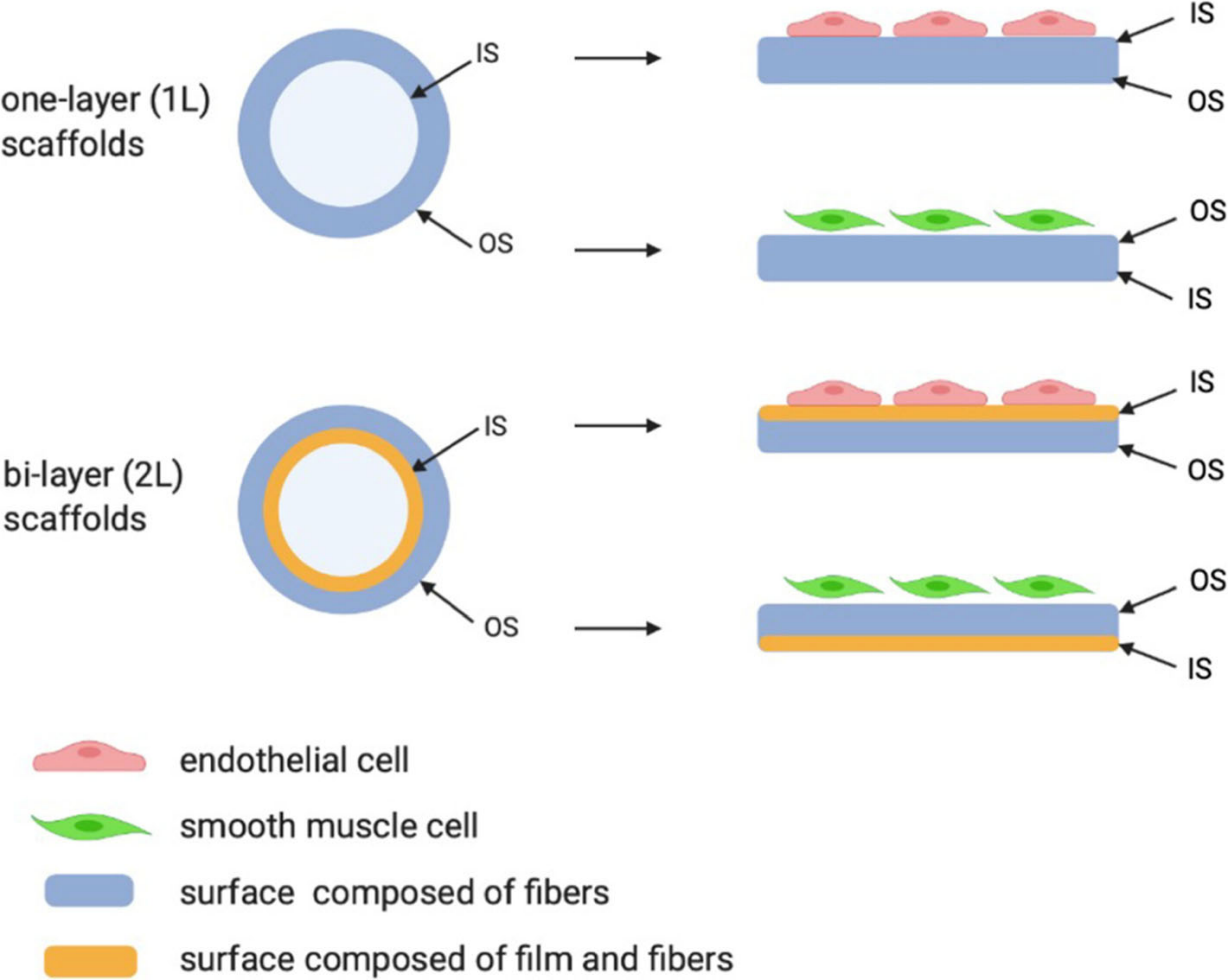
Scheme presenting the idea of the study. Blow spun scaffolds were manufactured using different process parameters, which resulted in obtaining two types of materials (1L and 2L). Next, two types of cells were cultured on the opposite surfaces of the scaffolds: endothelial cells (ECs) were seeded on the inner surface (IS), smooth muscle cells (SMCs) were seeded on the outer surface (OS). Created with BioRender.com

Sterile scaffolds were placed in the 24-well plates, mounted with inserts, and incubated with medium for 1 h at 37 °C. Cells (ECs or SMCs) were harvested, seeded on the material at the seeding density of 1 × 10^5^ cells/ml, and cultured at 37 °C in a 5% CO_2_ humidified atmosphere. Cultures with different types of cells were carried out on different surfaces of the scaffolds (Fig. 1). ECs were cultured on the inner surface of scaffolds (IS), whereas SMCs were cultured on the outer surface (OS).

#### Cell viability

ECs viability was tested using alamarBlue^TM^ assay (Thermo Fisher Scientific) according to the manufacturer’s protocol. Cells were seeded on materials’ surfaces as described above. Cells cultured in wells with no material (seeding density = 1 × 10^5^ cells/ml) were used as a control for viability calculation. After 1 d and 7 d of culture, the medium was removed from wells, and 500 µl of alamarBlue^TM^ working solution (alamarBlue^TM^ reagent 10x diluted in fresh DMEM without phenol red) was added (500 µl/well). Materials were incubated for 4 h at 37 °C in a 5% CO_2_ humidified atmosphere (protected from light). Following incubation, 100 µl of alamarBlue^TM^ solution was transferred in triplicate to a 96-well plate (black), and fluorescence (Ex. = 550 nm, Em. = 590 nm) was measured. Samples were washed with PBS (3x, 5 min on a plate shaker), incubated with fresh DMEM, and used for further viability measurements. Viability results are presented as the percentage of positive control according to the equation:

$$\mathrm{Viability}\;(\%)\;=\;(\mathrm{FI}\;{\mathrm{sample}}_{\mathrm t=\mathrm n}/\mathrm{FI}\;{\mathrm{control}}_{\mathrm t=1})\;\bullet\;100\%,$$

where:

FI sample – fluorescence intensity of the sample after “n” days of culture,

FI control - fluorescence intensity of the control after 1 day of culture.

#### LDH release

LDH release during ECs culture was evaluated using CyQUANT^TM^ LDH Cytotoxicity assay (Thermo Fisher Scientific) according to the manufacturer’s setup. ECs were seeded on the materials as described above. After 1 d, 3 d, and 7 d of culture, 50 µl MCDB-1 medium from over the material was transferred to 96-well plate triplicate, and LDH reagent (Substrate Mix) was added. After 30 min of incubation at room temperature, stop solution was added, and absorbance (490 nm and 680 nm) was measured. The medium was changed 24 h before the test each time. The LDH release was calculated as a percentage of LDH released from control, according to the equation:

$$\mathrm{LDH}\;\mathrm{release}\;(\%)\;=\;(\mathrm{Abs}\;{\mathrm{sample}}_{\mathrm t=\mathrm n}/\mathrm{Abs}\;{\mathrm{control}}_{\mathrm t=1})\;\bullet\;100\%,$$

where:

Abs sample – absorbance of the sample after “n” days of culture,

Abs control - absorbance of the control after 1 day of culture.

#### Collagen secretion

Collagen I alpha 1 ELISA Kit (ab210966, Abcam) was used to measure collagen secretion during SMCs culture. SMCs were seeded as described above. On the given day of culture (D1, D5, D7, D10, D14), the medium was collected from the materials, centrifuged at 2000 x g for 10 min, and diluted 1: 4 with Sample Diluent. Solutions were stored at -20 °C until samples from all time points were collected. The test was performed according to the manufacturer’s instructions. Briefly, 50 µl of the sample and 50 µl of the Antibody Cocktail were added to a well of a 96-well plate and incubated for 1 h at RT. After this time, each well was washed 3 × 350 µl 1X Wash Buffer. Then 100 µl of TMB Development Solution was added to each well, incubated in the dark for 10 min. Finally, 100 µl of Stop Solution was added to each well, and the plate was shaken for 1 min. Absorbance was measured at 450 nm. The amount of released collagen was calculated based on the standard curve equation prepared from the standards provided in the kit.

#### Cell adhesion

The number of surface-adhered cells was calculated using a confocal microscope (LSM 880, Zeiss) equipped with ZEISS ZEN software (Zen 2). After a given time of culture, samples were washed with PBS (4x, 5 min on a plate shaker) and fixed with 4% w/v paraformaldehyde (Sigma-Aldrich, 500 µl/well). Plates were incubated at 4 °C for 24 h and washed with PBS (3x, 5 min on a plate shaker). Then cells were permeabilized by adding 500 µl/well of 0.2% Triton X-100 for 8 min. Materials were washed with PBS (4x, 5 min on a plate shaker). Next, materials were incubated with AlexaFluor 488 (300 µl/well, ThermoFisher Scientific) for 1 h in the dark, washed with PBS (4x, 5 min on a plate shaker), and incubated with 300nM DAPI (300 µl/well, ThermoFisher Scientific) at room temperature for 6 min in the dark. Finally, samples were washed with PBS (4x, 5 min on a plate shaker), placed on microscope slides with a drop of glue (ProLong Diamond Antifade Mountant, Invitrogen), covered with cover slides and observed using the confocal microscope. Cell nuclei were stained with DAPI dye. Images in magnification 20x were taken for each type of sample (*n *= 6). Cell number per mm^2^ and cell coverage (calculated as the ratio of the area occupied by the cells to the area of the sample) were counted using Fiji software [28] based on the number of visible nuclei (cell number) or area of actin-stained cells (cell coverage). SMCs infiltration was calculated from CLSM images using Zeiss software and z-stacking function.

### Statistical analysis

Statistical significance of differences was analyzed using single-factor analysis of variance (ANOVA) for *p *< 0.05 with post hoc Tukey’s test (OriginPRO 2020b).

## Results

### Scaffold characterization

#### Morphology

Figure 1 summarizes the idea of the study. All materials were obtained in the form of cylindrical scaffolds in which the inner (collector side, IS) and outer (OS) surfaces were distinguished. Cells were grown on different sides of the scaffold surfaces, depending on the cells analyzed: ECs on the inner surface and SMCs on the outer surface. ECs were cultured on six types of material, differing in morphology (one-layer and bi-layer) and an average fiber diameter (designated as C75A_1L_200, C75A_1L_500 and C75A_1L_900, C75A_2L_200, C75A_2L_500 and C75A_2L_900). As the outer surface (OS) for 1L- and 2L-type materials were produced in the same way, SMCs were cultured only on three types of material differing in average fiber diameter (designated as C75A_200, C75A_500, and C75A_900).

Materials were made in two morphological variants: a one-layer (1L) and a bi-layer (2L) and Table 2 lists the material variants analyzed in the study. A homogeneous fibrous structure in cross-section characterized 1L-type materials - both surfaces (IS and OS) consisted of fibers of similar morphology. A structure variety in cross-section characterized 2L-type materials - the outer layer (OS) was made of fibers, while the inner surface (IS) was made of mixed fibrous and solid areas. Bi-layer scaffolds were obtained in one two-step SBS process. It was possible thanks to the use of the variable nozzle-collector working distance: shorter (10 cm) during the production of the first layer and longer (30 cm) during the production of the second layer. Additionally, three different polymer concentrations were used to obtain three different size groups of fibers within each material group. The concentrations of polymer were selected to obtain fibers with an average diameter in the range of 200-300 nm (1L_200, 2L_200), 500-600 nm (1L_500, 2 L_500), and 900-1000 nm (1L_900, 2L_900).

**Table 2:** Material variants analyzed in the study

Sample	Polymer solution concentration [% w/w]	Number of layers [-]	Inner surface (IS)	Outer surface (OS)
C75A_1L_200	2	1	fibers	fibers
C75A_1L_500	4	1	fibers	fibers
C75A_1L_900	5	1	fibers	fibers
C75A_2L_200	2	2	film/fibers	fibers
C75A_2L_500	4	2	film/fibers	fibers
C75A_2L_900	5	2	film/fibers	fibers

**Fig. 2:**
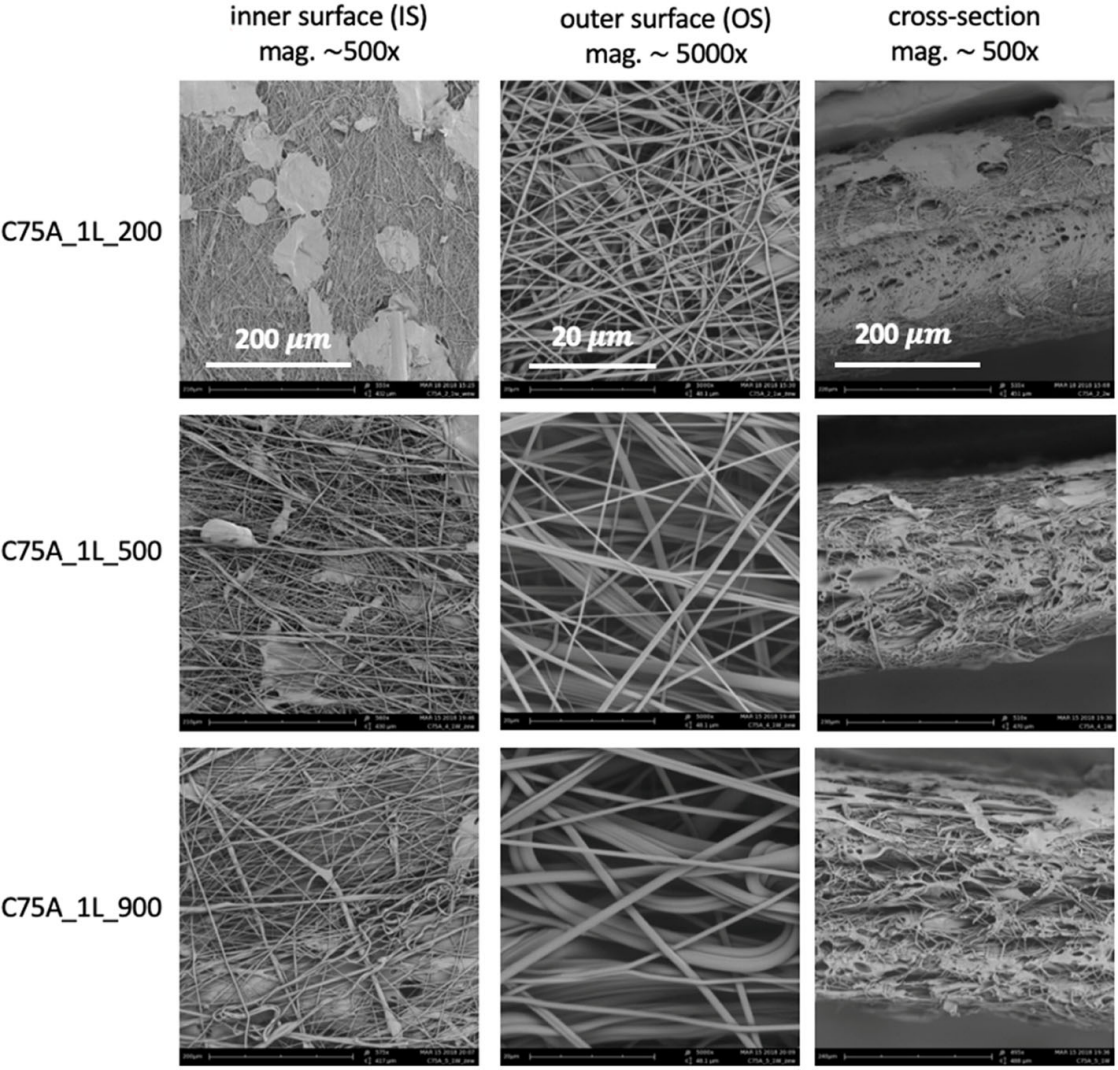
Morphology of one-layer (1L) scaffolds

**Fig. 3:**
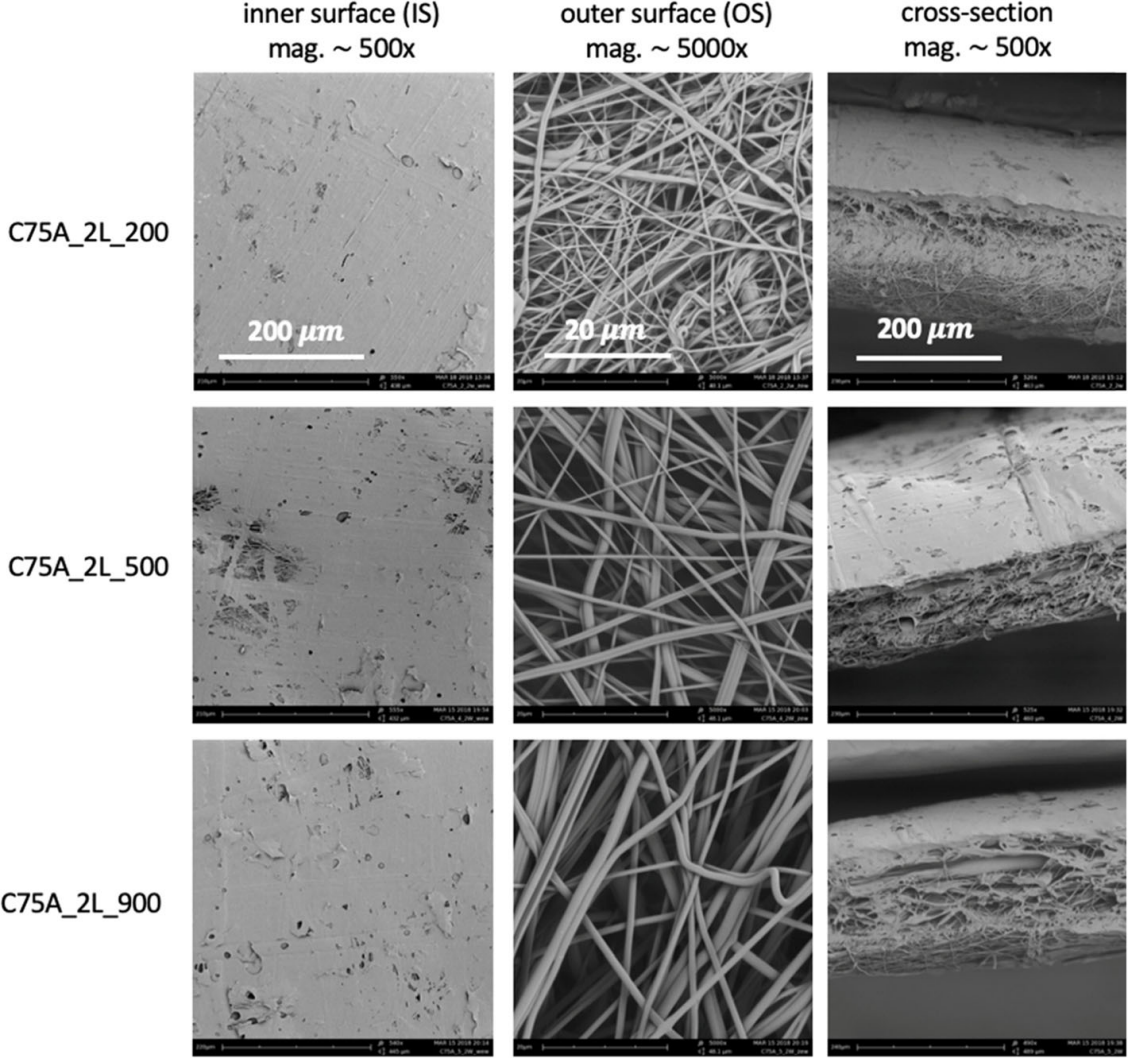
Morphology of bi-layer (2L) scaffolds

Figures 2 and 3 show the morphology of 1L-type and 2L-type materials. It was possible to produce fibers in the assumed diameter ranges: 200-300 nm for a polymer concentration of 2% w/w, 500-600 nm for a concentration of 4% w/w, and 900-1000 nm for a concentration of 5% w/w. In the case of 1L-type materials, the inner surface was characterized by the presence of local defects in the form of solid, non-fibrous areas. The number of such defects was higher on materials made from the lowest polymer concentration (C75A_1L_200). The number of defects on the outer surface of the materials was much smaller. Figure 2 also shows a cross-section of the obtained materials. It can be noticed that the materials show a homogeneous fibrous structure along the cross-section, while local defects in the form of solid areas appear on the inner surface. In the case of 2L materials (Fig. 3), the inner surface mostly showed a solid structure (film), with local areas composed of fibers with the average diameters depending on the polymer concentration used in the process. The outer surface was made of fibers, and it appears to have the same structure as both surfaces in the case of 1L-type materials. SEM images of the cross-sections show a change in the structure along the cross-section - on the inner surface, there is a thin layer of solid film, then the material changes into a fibrous structure. For both types of scaffolds (1L and 2L), the material thickness is similar, and it remains in the 300 - 500 μm range.

**Fig. 4:**
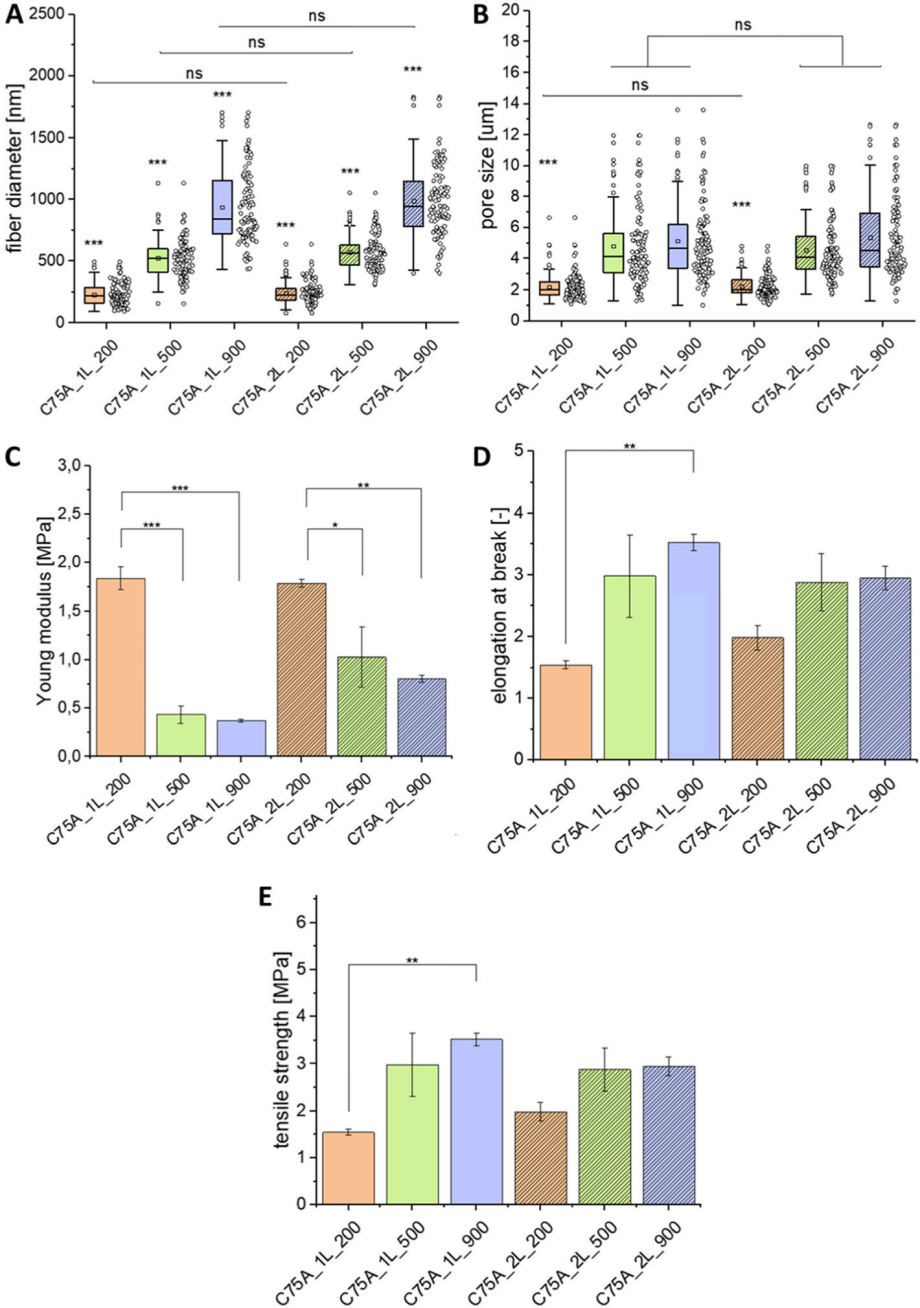
Fiber diameter (**A**) and pore size distribution (**B**). In the case of 2L-type materials, the values refer to the fibrous areas on the outer surface. ns – not significant, ***p < 0.001 vs. each other group. Mechanical properties of the analyzed materials: Young's modulus (**C**), elongation at break (**D**), and tensile strength (**E**). MV±SD, *n *= 5, *p < 0.05, **p <0.01, ***p <0.005

Figure 4A presents histograms of fiber diameter distribution. As expected, the average fiber diameter increases as the polymer solution concentration increases. Also, the average fiber diameter obtained for the different polymer solution concentrations was statistically different (*p *< 0.001). For one-layer structures, these values are respectively: 232 ± 89 nm (C75A_1L_200), 524 ± 15 nm (C75A_1L_500) and 936 ± 302 nm (C75A_5_1L_900). The average fiber diameters obtained for bi-layer structures are similar: 245 ± 90 nm (C75A_2L_200), 572 ± 140 nm (C75A_2L_500), 987 ± 287 nm (C75A_2L_900). No statistically significant differences were found between the average fiber diameter for analogous materials from the 1L- and 2L-type group.

As the polymer solution concentration increases, the values of minimum (d_min_) and maximum (d_max_) diameter also increases. Narrow diameter distributions were obtained from the polymer solution with the lowest concentration (2% w/w). This result was obtained for both, one-layer and bi-layer structure. d_min_ values were respectively 97 nm (C75A_1L_200) and 81 nm (C75A_2L_200). d_max_ values were 493 nm (C75A_1L_200) and 636 nm (C75A_2L_200) respectively. Samples obtained from a 4% w/w polymer solution were characterized by a wider range of fiber diameters. d_min_ values were 158 nm (C75A_1L_500) and 310 nm (C75A_2L_500). d_max_ values were 1133 nm (C75A_1L_500) and 1054 nm (C75A_2L_500). The widest diameter distribution was obtained for the material obtained from a 5% w/w polymer solution. d_min_ values were 434 nm (C75A_1L_900) and 399 nm (C75A_2L_900). d_max_ values were 1705 nm (C75A_1L_900) and 1829 nm (C75A_2L_900).

Figure 4B presents pore size distribution. There were no significant differences between one- and bi-layer structures. As expected, the average pore size increases as the average fiber diameter increases, however only for the group with the lowest average fiber diameter, the average pore size was significantly different from other groups (*p *< 0.001). For materials with an average fiber diameter of about 200 nm, the average pore size was approximately 2 μm (C75A_1L_200: 2.2 ± 0.9 μm, C75A_2L_200: 2.2 ± 0.7 μm). For these materials, the minimum pore size was slightly above 1 μm (C75A_1L_200: 1.1 μm, C75A_2L_200: 1.1 μm). The maximum pore size was less than 10 μm (C75A_1L_200: 6.6 μm, C75A_2L_200: 4.8 μm). For materials with average fiber diameter in the 500-600 nm range, the average pore size was approximately 5 μm (C75A_1L_500: 4.8 ± 2.5 μm, C75A_2L_500: 4.6 ± 1.9 μm). The minimum pore size was above 1 μm (C75A_1L_500: 1.3 μm, C75A_2L_500: 1.7 μm).

The maximum pore size has exceeded the value of 10 μm (C75A_1L_500: 12.0 μm, C75A_2L_500: 10.0 μm). Similar pore size values were obtained for materials with the highest average fiber diameter (900-1000 nm). The average pore size or those materials was also approximately 5 μm (C75A_1L_900: 5.2 ± 2.4 μm, C75A_2L_900: 5.4 ± 2.6 μm). The minimum pore size was slightly above 1 μm (C75A_1L_900: 1.0 μm, C75A_2L_900: 1.3 μm). The maximum pore size exceeded the value of 10 μm (C75A_1L_900: 13.6 μm, C75A_2L_900: 12.7 μm).

#### Surface wettability

The results of water contact angle (WCA) measurement are shown in Table 3. All analyzed surfaces were hydrophobic (WCA>90°).

**Table 3:** Water contact angle values measured for inner (IS) and outer (OS) surfaces of the materials. MV±SD, *n *= 30

Sample	OS water contact angle [°]	IS water contact angle [°]
C75A_1L_200	122 ±2	109±3
C75A_1L_500	127±4	121±3
C75A_1L_900	130±3	120±6
C75A_2L_200	123±4	91±5
C75A_2L_500	121±7	100±4
C75A_2L_900	125±3	109±14

The outer surface (OS) showed similar wettability values for all tested variants (above 120°). There were no statistically significant differences in the WCA values for analogous materials from the 1L- and 2L-type groups. This was expected since the outer surface is produced in the same way in both groups. There was no relationship between the value of WCA and the average diameter of the fibers building the surface - similar values were obtained for both surfaces composed of micron and submicron fibers.

In the inner surface (IS) case, higher WCA values were obtained for 1L-type scaffolds than the 2L-type. However, the differences were not statistically significant (*p *> 0.05).

For each analyzed material, the WCA values obtained for IS were lower than those obtained for OS.

#### Mechanical properties

The results of mechanical properties analysis are shown in Fig. 4. Materials were analyzed in the form of cylinders with an inner diameter of 3 mm. It has been shown that both the morphology type and fiber diameter affect the mechanical properties of the materials. In both cases, 1L-type and 2L-type materials, the Young's modulus (Fig. 4C) value decreased with an increase of average fiber diameter. The highest Young's modulus value was obtained for materials composed of fibers with the lowest diameter (1.8 ± 0.3 MPa for C75A_1L_200, 1.9 ± 0.2 MPa for C75A_2L_200). The values were statistically significantly different (*p *< 0.05) from the values obtained for other materials.

An inverse relationship was observed for the elongation at break values (Fig. 4D). Higher values were obtained for materials with higher average fiber diameter. Comparison of 1L-type and 2L-type materials shows that in most cases, values of elongation are higher for 1L-type materials. The exceptions are materials with the smallest average fiber diameter (C75A_200), where this relationship was inverse. The elongation values are about 300% for materials with average fiber diameter in the 500-1000 nm range and about 150% for materials with average fiber diameter in the 200-300 nm range.

A similar relationship presented itself for the tensile strength values (Fig. 4E). The values increased with the increase in the fiber’s average diameter. This relationship was observed in both the 1L- and 2L-type groups. The highest values were obtained for materials composed of micron fibers (3.5 ± 0.3 MPa for C75A_1L_900, 3.1 ± 0.4 MPa for C75A_2L_900). There were no statistically significant differences in the tensile strength values between the analogous materials from the 1L- and 2L-type groups.

### Endothelial cells growth

#### Endothelial cells viability

**Fig. 5:**
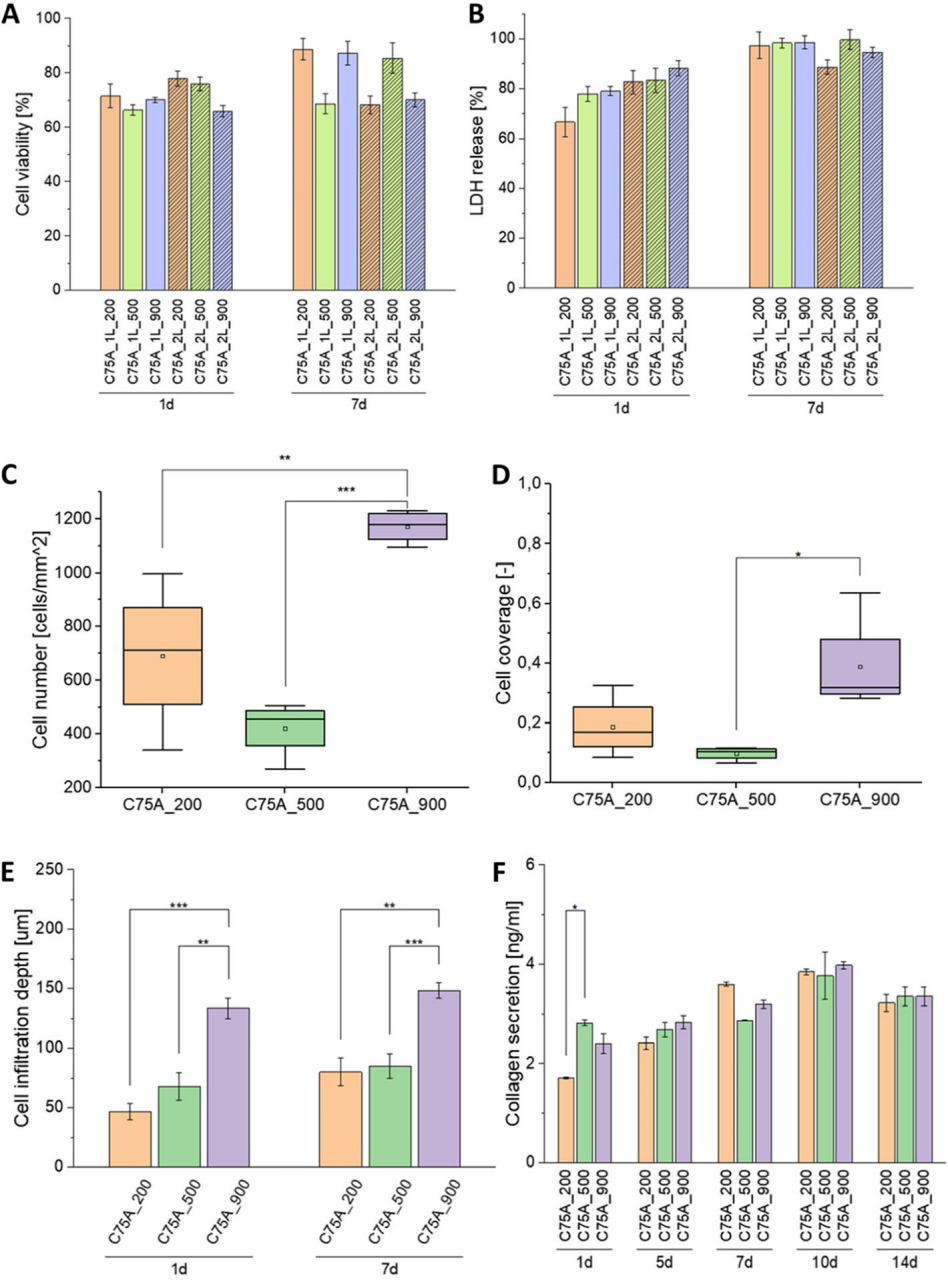
Endothelial cell viability (**A**) and LDH assay (**B**) after 1 day and 7 days of ECs culture. MV±SD, *n *= 3. Smooth muscle cell number (**C**) and cell coverage (**D**) obtained on the outer surface (OS) of the analyzed materials after 7 days of SMCs culture. SMCs infiltration depth after 1 and 7 days of culture (**E**). Collagen secretion after 1, 5, 7, 10 and 14 days of SMCs culture (**F**). MV±SD, *n *> = 3, *p < 0.05, **p < 0.01, ***p < 0.005

ECs viability (Fig. 5A) after 1 day of culture on scaffolds’ inner surface (IS) was similar for all types of analyzed samples. The viability values ranged from 66% (C75A_2L_900) to 78% (C75A_2L_200). No relationship between surface morphology and the viability of the cells was observed.

After 7 days of culture, cell viability for most tested materials increased compared to the 1 day of culture. Only for C75A_2L_200, the viability decreased. The viability values were in the range from 68% (C75A_2L_500) to 89% (C75A_2L_200).

After 1 day of culture, the amount of LDH released by cells growing on materials was lower than the amount of LDH released by the control (Fig. 5B). The lowest values were obtained for the 1L-type materials. The percentage of LDH release was in the range of 60-80%. In the case of 2L-type materials, this ratio was >80%.

After 7 days of culture, the amount of released LDH was on a similar level for all 1L-type materials, and the values were slightly below 100%. For the 2L-type materials, the LDH release was, in most cases, smaller; however, the differences were not statistically significant (*p *> 0.05).

#### Endothelial cells adhesion

**Fig. 6:**
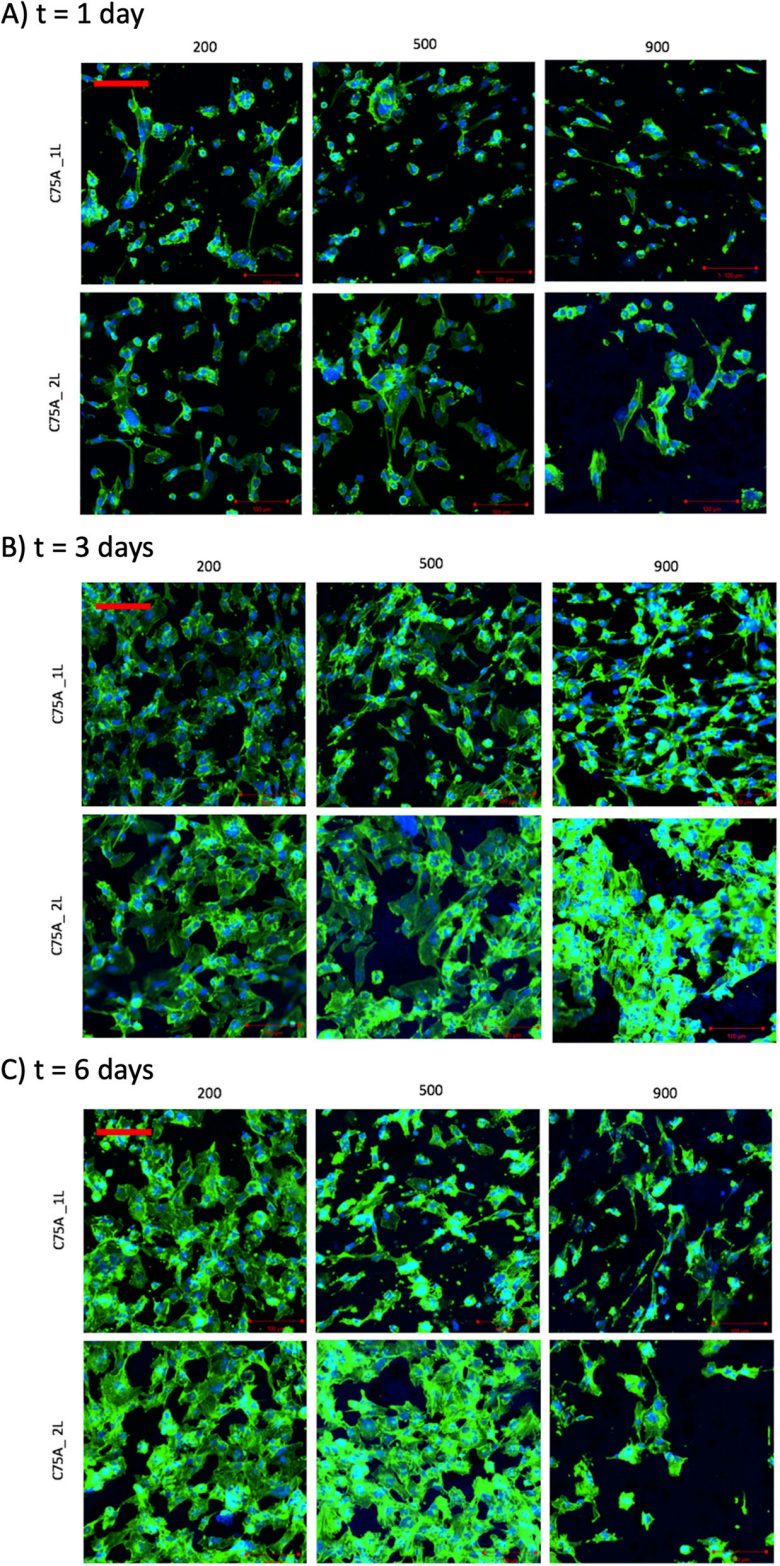
ECs adhesion on the inner surface (IS) of the analyzed materials after 1 (**A**), 3 (**B**), and 6 (**C**) days of culture. Scale bar: 100 μm

**Fig. 7:**
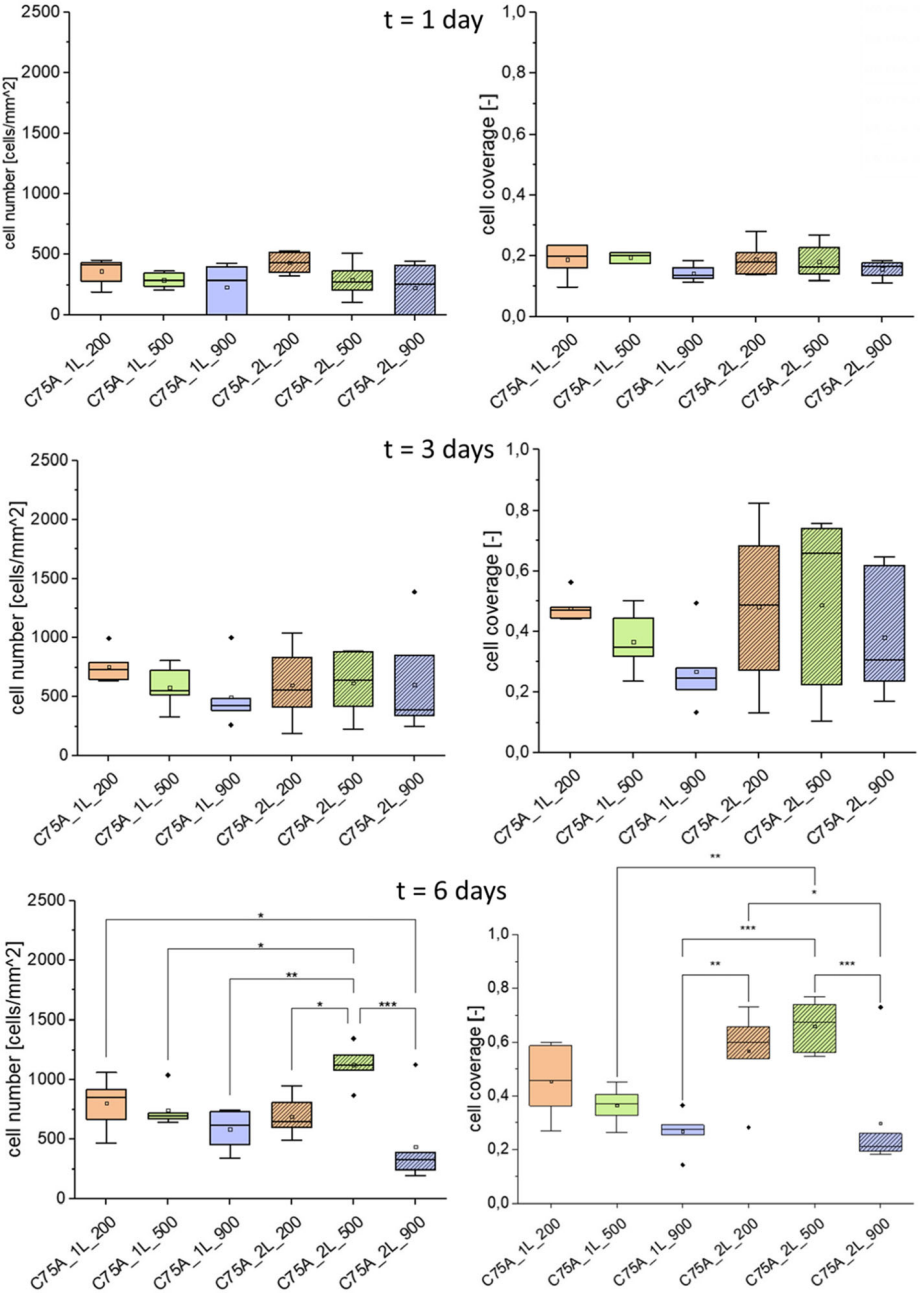
Endothelial cell number and cell coverage obtained on the analyzed materials’ inner surface (IS) after 1, 3, and 6 days of ECs culture. MV±SD, *n *= 6, *p < 0.05, **p < 0.01, ***p < 0.005

Figure 6 shows ECs growing on the inner surface (IS) of the analyzed materials after 1, 3, and 6 days of culture. The number of cells per mm^2^ and cell coverage are shown in Fig. 7.

After 1 day of culture, all analyzed materials presented surface-adhered cells with normal morphology. Cell growth was homogeneous – there were no areas with significantly fewer or more cells noticed. In the case of 1L-type materials, the number of cells per mm^2^ decreased with the increase of the average fiber diameter and was: 364 ± 103 cells/mm^2^ for C75A_1L_200, 290 ± 63 cells/mm^2^ for C75A_1L_500 and 233 ± 188 cells/mm^2^ for C75A_1L_900 respectively. The same relationship was observed for 2L-type materials. The number of cells per mm^2^ was: 431 ± 86 cells/mm^2^ for C75A_2L_200, 290 ± 139 cells/mm^2^ for C75A_2L_500, and 227 ± 193 cells/mm^2^ for C75A_2L_900 respectively. Again, the differences between the individual material variants were not statistically significant (*p *> 0.05). The values of cell coverage were similar for all tested surface variants and were smaller than 0.2. In the case of 1L-type materials, they were 0.19 ± 0.05, 0.19 ± 0.02, 0.14 ± 0.03 respectively for C75A_1L_200, C75A_1L_500, and C75A_1L_900. For 2L-type materials, the cell coverage was similar: 0.19 ± 0.05, 0.18 ± 0.06, 0.16 ± 0.03 for C75A_2L_200, C75A_2L_500, and C75A_2L_900 respectively.

After 3 days of culture, the number of cells per mm^2^ increased for all materials. On 1L-type materials, there was a decrease in the number of cells and the cell coverage with the increase in the average fiber diameter clearly visible. The number of cells per mm^2^ was: 757 ± 131 cells/mm^2^, 580 ± 169 cells/mm^2^, 497 ± 258 cells/mm^2^ corresponding to C75A_1L_200, C75A_1L_500, and C75A_1L_900. The cell coverage was respectively: 0.48 ± 0.04, 0.37 ± 0.09, and 0.27 ± 0.12 for C75A_1L_200, C75A_1L_500, and C75A_1L_900. The relationship was different for 2L-type materials. The number of cells per mm^2^ was similar for all three types of materials and amounted to: 600 ± 313 cells/mm^2^, 617 ± 289 cells/mm^2^, 603 ± 439 cells/mm^2^, respectively for C75A_2L_200, C75A_2L_500, and C75A_2L_900. The values of cell coverage were also similar for all materials but showed great differentiation - there were areas with a large number of cells as well as areas without them. The values of cell coverage were slightly below 0.5 and amounted to: 0.48 ± 0.26, 0.49 ± 0.28, and 0.38 ± 0.20 for C75A_2L_200, C75A_2L_500, and C75A_2L_900, respectively.

After 6 days of culture, the differences in cell growth on individual materials were clearly visible. The highest cell coverage was obtained on the surfaces C75A_2L_200 (0.57 ± 0.15) and C75A_2L_500 (0.66 ± 0.09). Microscopic observations confirmed uniform cell growth on these surfaces. On 1L-type surfaces, the decrease in cell number and cell coverage with the increase of average fiber diameter was still maintained. The values of the number of cells per mm^2^ were: 804 ± 208 cells/mm^2^, 744 ± 147 cells/mm^2^, 585 ± 158 cells/mm^2^ for C75A_1L_200, C75A_1L_500, and C75A_1L_900, respectively. The cell coverage was: 0.46 ± 0.13, 0.37 ± 0.07, and 0.27 ± 0.07 for C75A_1L_200, C75A_1L_500, and C75A_1L_900, respectively.

### Smooth muscle cells growth

#### Smooth muscle cells adhesion and infiltration

**Fig. 8:**
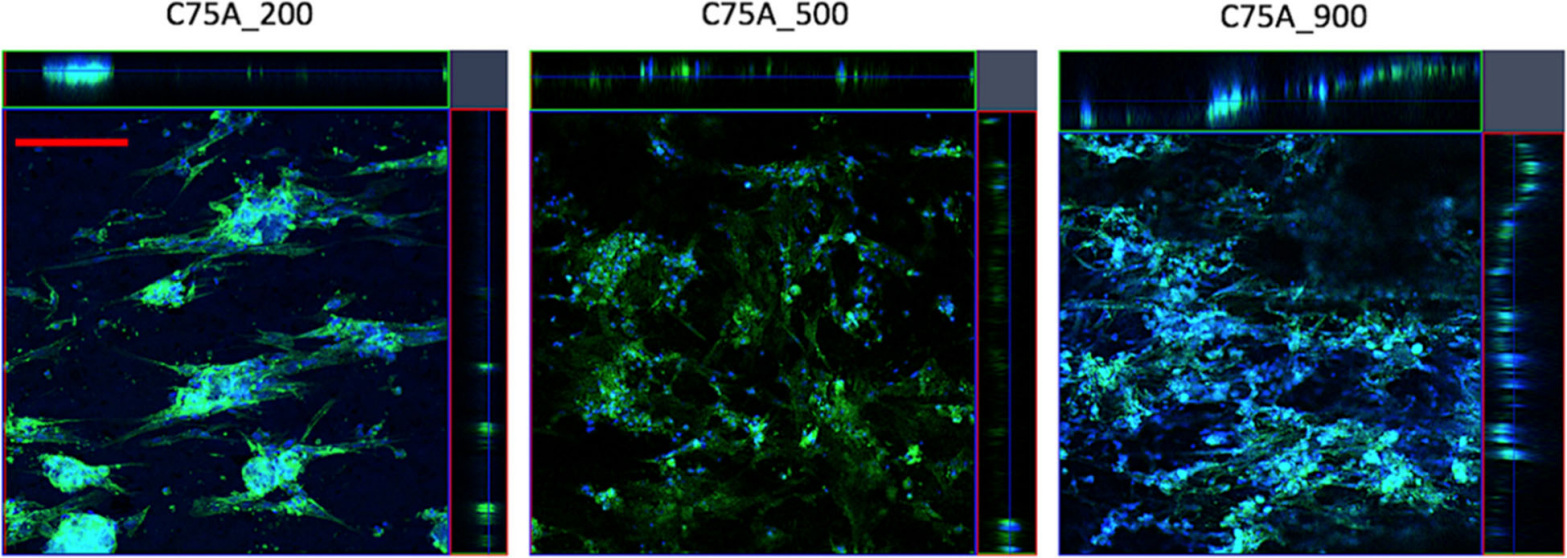
SMCs adhesion after 7 days of culture on the analyzed materials’ outer surface (OS). Scale bar: 100 μm

Figure 8 shows SMCs growing on the outer surface (OS) of the analyzed materials after 7 days of culture. The number of cells per mm^2^ and cell coverage are shown in Fig. 5C and D. As the outer surface (OS) for 1L- and 2L-type materials were produced in the same way, SMC culture was performed only for materials differing in average fiber diameter (designated as C75A_200, C75A_500, and C75A_900).

Analysis of SMCs growth on the materials’ outer surface (OS) after 7 days of culture showed a significantly higher number of cells on the C75A_900 materials. The number of cells per mm^2^ was: 691 ± 270 cells/mm^2^, 422 ± 104 cells/mm^2^ and 1172 ± 61 cells/mm^2^ for C75A_200, C75A_500, and C75A_900, respectively. The cell coverage values were: 0.19 ± 0.10, 0.10 ± 0.02, and 0.39 ± 0.17 for C75A_200, C75A_500, and C75A_900, respectively.

Figure 5E shows infiltration depth after 1 and 7 days of culture. Both after 1 and 7 days of culture, the infiltration depth was significantly higher for the C75A_900 material and amounted to 133 ± 15 μm and 148 ± 16 μm after 1 and 7 days of culture, respectively. For each of the tested materials, the infiltration depth after 7 days of culture was greater than the value achieved after 1 day.

#### Collagen secretion

The analysis of collagen secretion during the 14-day culture showed that during the first 10 days, the amount of secreted collagen increased for all analyzed material variants (Fig. 5F). On the 14th day of culture, the amount of collagen decreased compared to day 10. On individual days, the differences between the material variants were minor and, in most cases, were not statistically significant. Only on the first day of culture, statistically significantly higher (*p* <0.05) collagen secretion was obtained on the C75A_500 material (2.82 ± 0.08 ng/ml) vs. C75A_200 (1.72 ± 0.02 ng/ml). In the case of all analyzed materials, the highest values of secreted collagen were achieved on the 10th day of culture, and they were: 3.84 ± 0.08 ng/ml, 3.76 ± 0.67 ng/ml, and 3.98 ± 0.11 ng/ml, respectively, for C75A_200, C75A_500, and C75A_900.

## Discussion

The presented work aimed to determine the influence of the structure of fibrous scaffolds on the growth of two types of cells building blood vessels: ECs and SMCs. The materials were produced by the SBS technique using a medical-grade polyurethane solution. Design of the production process aimed at fabrication of one-layered and bi-layered cylindrical scaffolds in one process, advantageous to other – two-step approaches [31, 32]. The produced materials in the form of cylinders were cut open, and two surfaces were separately analyzed. The growth of ECs was analyzed on the inner (collector side) surface, whereas the SMCs growth was analyzed on the outer surface.

Two types of material were prepared, differing in the morphology of the inner surface and the cross-section structure. The first type were fibrous materials of homogenous cross-section structure, designated as one-layer (1L). Scaffolds were produced with a constant nozzle-collector working distance of 30 cm. The second type of materials presented variable structure. The inner surface consisted of non-fibrous (solid) areas and fibrous areas. Such a structure was obtained by reducing the nozzle-collector working distance (10 cm) during the production of the first inner layer. The second, fibrous outer layer, was produced with an increased nozzle-collector distance (30 cm). In this way, materials of variable structure, designated as bi-layer (2L), were obtained. Within each type, 3 groups of materials were produced, differing in the range of fiber and pore size. The first group obtained from the 2% w/w polymer solution was characterized by an average fiber diameter in the range of 200-300 nm and average pore size of approximately 2 μm. The second group obtained from the 4% w/w solution was characterized by an average fiber diameter in the range of 500-600 nm and average pore size of approximately 5 μm. The third group obtained from a 5% w/w solution was characterized by an average fiber diameter in the range of 900-1000 nm and average pore size of approximately 5 μm. This evident influence of polymer solution concentration on fiber size and, consequently, on pore size was expected, as previously reported in the body of research concerning the solution blow spinning process [33–36]. Dependence of average fiber diameter on polymer concentration in solution allowed for the design of scaffolds for this study.

The culture of ECs was carried out for a week with observations after 1, 3, and 6 days of culture. The results showed that cell growth was similar on all types of materials after the first day of culture, regardless of the morphology. The fraction of the area occupied by the cells was approximately 0.2. After 3 days of culture, the number of cells on all materials increased, which confirmed the proper cell growth. There was also variation in the surface area occupied by cells depending on the type of material. In the group of 1L-type materials, a decrease in the number of cells was observed, along with an increase in the average fiber diameter. The same relationship was observed for the cell coverage. However, in the group of 2L-type materials, there was no difference in the number and cell coverage depending on the average diameter of the fibers. What is more, it was observed that the growth of cells was uneven - large standard deviations characterized both tested parameters. Microscopic analysis showed that the cells were mainly growing on fiber-free areas composed of a solid polymer film. The fibrous areas were characterized by a much smaller number of cells. After 6 days of culture on 1L-type materials, the relationship mentioned earlier was maintained - both the mean number of cells and the cell coverage increased as the average diameter of the fibers decreased. Greater differentiation appeared in the group of 2L-type materials. Statistically significant differences were obtained in both the number of cells and the cell coverage. The highest values were obtained for materials with average fiber diameter in the range of 200-300 nm and 500-600 nm. For these materials, the values of cell coverage exceeded 0.6. For 2L-type materials with the largest average fiber diameter (900-1000 nm range), the smallest cell coverage (approximately 0.2) was observed.

In the case of SMCs, the growth on the surface marked as outer was analyzed. It was assumed that this surface should be fibrous to promote SMCs infiltration and blood vessel formation. Therefore, in the case of the outer surface, mixed structures composed of solid and fibrous areas were not analyzed. As in the case of ECs, the growth of SMCs was investigated on surfaces with an average fiber diameter ranging from 200 to 300 nm, 500-600 nm, and 900-1000 nm. The results clearly indicated that the best growth after 7 days of culture was obtained on the fibers with the largest diameters studied (900-1000 nm). The highest cell infiltration depth was also obtained for these materials, which can be attributed to the largest pore sizes among tested materials. At the same time, there were no significant differences in the amount of collagen secreted by SMCs growing on fibers of different diameters.

The study showed that the morphology of the materials significantly influences the rate of monolayer formation by ECs. The study aimed to select the inner surface of cylindrical blow spun scaffolds to obtain a fast rate of endothelial monolayer formation. In particular, the research aimed to compare one-layer structures made of fibers alone with bi-layer structures made of fibrous and solid areas.

Many studies show that cell growth is strongly dependent on the surface roughness, and in the case of fibrous materials, on the average diameter of the fibers. The nature of this relation depends on the type of cells. In the case of ECs growing on the surface of vascular prostheses, the main goal is to increase adhesion and create a monolayer on the prosthesis surface without the cells infiltrating the material. It has been shown that the increase of roughness at the nanoscale promotes adhesion of ECs [37]. A similar study was carried out for polylactide materials, which showed better growth of ECs on solid surfaces than fibrous ones [38]. Xu et al. suggests that the best solution in the case of vascular prostheses may be mixed structures created by combining solvent casting and electrospinning techniques. Most studies suggest better adhesion of ECs to nanofibers compared to microfibers. However, there are studies indicating the opposite relationship [39].

In the case of SMCs growth, many studies show that better growth is achieved on surfaces with higher roughness and larger average fiber diameter. Ju et al. showed that SMCs need fibers with an average diameter > 1 μm for proper development and infiltration [12]. Han et al. showed that increasing the fiber diameter increases the infiltration of SMCs but reduces their proliferation [14].

It is worth mentioning that the morphology, particularly the roughness and the size of the fibers, also has a significant impact on the adhesion of blood components, particularly platelets [40], which is of crucial importance in the case of vascular prostheses. That is why it is essential to select the appropriate morphology in the design of modern, biocompatible vascular prostheses. The presented results clearly indicate that in the case of fibrous vascular prostheses, multilayer structures composed of fibers of various sizes and solid non-fibrous areas are a promising solution. We proved that the best growth of SMCs is obtained for micron fibers (with an average diameter in the 900-1000 nm range) compared to the submicron fibers (with an average diameter below 900 nm). In the case of ECs, it seems that mixed structures composed of fibrous and solid areas are the best solution. Therefore, the best solution is a prosthesis consisting of a microfiber outer layer and a mixed inner layer containing fibrous and solid areas. Such a mixed, multilayer structure can be obtained using the SBS technique by appropriate selection of the process parameters (concentration of the polymer solution, working distance of the nozzle-collector). The solid regions promote the adhesion of ECs and accelerate the formation of the monolayer. On the other hand, the outer surface composed of micron fibers promotes the growth and infiltration of the SMCs.

## Conclusions

Fibrous structures with different morphology were obtained using SBS technique. The materials differed in the average diameter of the fibers and the number of layers. The study investigated the effect of surface morphology on the growth of ECs and SMCs. In the case of ECs, we compared cell growth on fibrous surfaces (1L-type) with different average fiber diameters and mixed surfaces (2L-type) composed of solid and fibrous areas. ECs showed a higher cell coverage on mixed surfaces. These differences between surfaces became clearly visible after 6 days of culture. In the case of SMCs, better growth was shown on micron fibers compared to submicron fibers.

## Data Availability

Please contact author for data requests.

## Abbreviations

EC: Endothelial cells, ES - electrospinning
IS: Inner surface
OS: Outer surface
SBS: Solution blow spinning
SMC: Smooth muscle cells
